# Delayed Cardiac Metastasis from Renal Cell Carcinoma Caused by *VHL* Mutation

**DOI:** 10.15586/jkcvhl.v10i1.258

**Published:** 2023-02-07

**Authors:** Christopher L. Sudduth, Anthony Castagno, Peter Maggs

**Affiliations:** Department of Cardiac Surgery, Mount Auburn Hospital, Harvard Medical School, Cambridge, MA, USA

**Keywords:** cardiac, metastatic, mutation, renal cell carcinoma, VHL

## Abstract

Cardiac metastasis caused by renal cell carcinoma (RCC) without vena caval involvement is rare. No mutation has been associated with this unique phenotype. A 77-year-old male presented to our clinic with a symptomatic right ventricular mass after nephrectomy for clear cell RCC (ccRCC). The mass was resected, and metastatic disease was confirmed. Targeted exon sequencing identified a *VHL* mutation (c.494T > G, p.V165G) in the resected specimen. While more than half of ccRCC cases are associated with *VHL* mutations, this case is the first to show the association between delayed, isolated cardiac metastasis and *VHL* V165G mutation. The phenotype presented 12 years after nephrectomy and localized to the right ventricular apex. Further genomic characterization of cases with cardiac metastases may provide clues regarding unique mutations noted. Patients exhibiting delayed spread of RCC to the heart must be screened for this mutation.

## Introduction

Isolated cardiac metastases from renal cell carcinoma (RCC) is extremely rare (1). Most cardiac metastases are found in the setting of diffuse systemic diseases. So far, only 16 cases of isolated cardiac metastases have been reported (2). The prognosis of these patients is poor. Large trials are not possible because the condition is uncommon. The majority of RCC cases are of clear cell histological subtype. Over half (57%) of them are associated with loss-of-function mutations in the Von Hippel–Lindau (*VHL*) tumor suppressor gene (3). Mutations in *PBRM1, SETD2, BAP1*, and *MTOR* genes also have been described (4). The variant associated with delayed cardiac metastasis is, however, unknown. The purpose of this study was to identify the cause of delayed isolated cardiac metastasis due to RCC.

## Case Report

**Figure 1:**
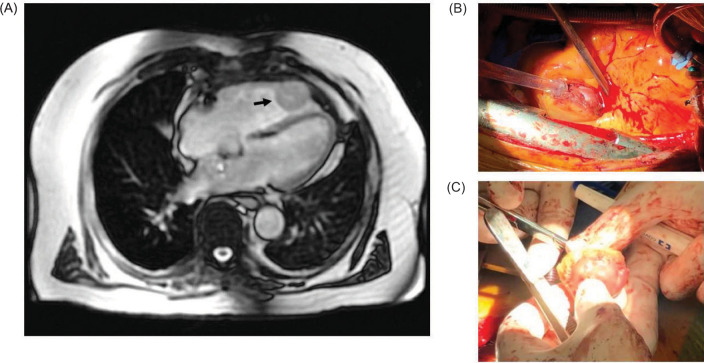
A 77-year-old male with delayed isolated cardiac metastasis from renal cell carcinoma; (A) Computed tomography demonstrating an enhancing, circumscribed T2 hyperintense T1 isointense right ventricular mass at the ventricular apex measuring 3.5 × 3.3 cm (arrow); (B) Intraoperative image showing the cardiac metastasis identified in the right ventricular wall; (C) Intraoperative image showing the final resected specimen sent for targeted sequencing. A VHL mutation was identified c.494T > G (p.V165G) in exon 3 in 41% of 241 reads.

Informed consent was obtained in accordance with the Office of Research. A 65-year-old male presented with an incidental finding of a right renal mass. He underwent right radical nephrectomy, and pathology showed clear cell RCC (ccRCC) pT3a with negative margins. The nephrectomy specimen was not sent for genomic testing. Twelve years later, he presented with progressive dyspnea and unintentional weight loss. Computed tomography and echocardiogram demonstrated a right ventricular apical mass measuring 3.5 × 3.3 cm in size (Figure 1). He underwent right ventricular tumor resection. Intraoperatively, the tumor was found to be attached to the right ventricular free wall, which was excised through circumferential excision. Pathology showed metastatic ccRCC. Immunohistochemistry was positive for PAX8, RCC antigen, and CD10, whereas it was negative for CK7, CK20, CD117, and epithelial membrane antigen (EMA).

The resected specimen underwent targeted exome sequencing with OncoPanel (Brigham and Women’s Hospital Department of Pathology, Boston, MA). The OncoPanel assay surveys exonic DNA sequences of 447 cancer genes and 191 regions across 60 genes for rearrangement detection. DNA was isolated from tissue containing at least 20% tumor nuclei and analyzed by massively parallel sequencing using solution-phase Agilent SureSelect hybrid capture kit and Illumina HiSeq 2500 sequencer. The specimen generated 14,209,117 aligned, high-quality reads with a mean 296 reads across all targeted exons, and 98% of all exons had more than 30 reads.

The ccRCC cardiac metastasis contained a *VHL* mutation c.494T > G (p.V165G) in exon 3 in 41% of 241 reads. Copy number analysis detected a concurrent single-copy deletion of the *VHL* gene (loss of 3p).

Postoperatively, the patient was managed with surveillance echocardiogram every 6 months. Combination therapies including tyrosine kinase inhibitors and immune checkpoint inhibitors (ICI) were considered; but, given the slow growth of the patient’s ventricular metastasis and considering the risks and benefits, it was decided not to proceed with these therapies. At 1-year follow-up, the patient was doing well with no symptoms and no evidence of recurrent disease.

## Discussion

A mutation in the *VHL* gene (V165G) can cause RCC with delayed cardiac metastasis following nephrectomy. The phenotype presented 12 years after nephrectomy and localized to the right ventricular apex. Most commonly, cardiac spread of RCC occurs due to direct tumor thrombus extension into the inferior vena cava (IVC). It rarely presents in the absence of IVC involvement. The median time for development is 8 years (2). Only 25% of the patients will present with asymptomatic disease; the most common symptoms are hypertension, dyspnea, arrhythmia, heart failure, chest pain, and hemodynamic instability (2).

Because isolated cardiac metastasis of RCC is an extremely rare event, detailed outcomes comparing resection alone or in combination with adjuvant or neoadjuvant targeted molecular therapies are lacking. Data from case series and isolated case reports suggest that isolated cardiac metastases are most commonly treated with resection alone (Table 1), while multifocal disease can be treated with medical therapy or by combination of both (2). Medical therapy most commonly includes vascular endothelial growth factor (VEGF)-targeted therapy. More recently, ICIs (nivolumab and ipilimumab) demonstrated efficacy in a single case report of a patient with cardiac metastasis (5). Neoadjuvant treatment in the setting of delayed isolated cardiac metastasis has not been reported.

**Table 1:** Review of the literature identified 4 reports of delayed presentation of isolated cardiac metastasis from renal cell carcinoma.

Author	Year	Patients (#)	Location of metastasis	Symptoms	Interval between RCC and metastasis (years)	Treatment
Aburto et al. (1)	2009	1	Left ventricle	Dyspnea	18	Resection alone
Talukder et al. (10)	2010	1	Left ventricle	Weight loss	22	Resection alone
Zhang et al. (11)	2013	1	Left ventricle	Asymptomatic	20	Sunitinib (inoperable tumor)
Viteri Malone et al. (2)	2018	13	Right ventricle (most common)	Variable	7.7(median)	Resection alone (most common)
Sudduth et al.	2023	1	Right ventricle	Dyspnea	12	Resection alone

RCC, renal cell carcinoma.

*VHL* is a tumor suppressor gene involved in the regulation hypoxia-inducible factors 1α and 2α (HIF-1α and HIF-2α). Biallelic inactivation of *VHL* causes upregulation of HIF target genes. This results in aberrant regulation of angiogenesis, apoptosis, and glycolysis. Recurrent mutations in *VHL* have been reported in 50–60% of ccRCC cases and are likely driver events in tumorigenesis (3, 6). The V165G mutation identified in our patient has not been reported previously. This missense mutation affects the elongin-binding region, altering the tumor suppression function of the *VHL* protein complex (7).

A genotype–phenotype correlation caused by two different missense mutations in the same codon in *VHL* has precedent: the Y112H mutation causes pheochromocytoma, while the Y112N mutation is more likely to cause RCC (8). Other missense mutations have also been reported at this position in RCC (V165D, 5 patients; V165I, 1 patient; V165L, 1 patient) (COSMIC). Phenotypic information was not provided for these cases.

Because RCC cardiac metastasis without IVC involvement is such a rare event, we hypothesized that it might be due to a mutation in a gene other than *VHL*. For the first time, we now know that a missense mutation in *VHL* is associated with delayed cardiac metastasis. This mutation may predispose RCC cells to develop a “second hit” mutation in another gene that causes preferential cardiac spread. The most common “second hit” mutations in RCC were tested (*SETD2, KDM5C, BAP1*, and *PBRM1*) (9) and were found negative. The mutation may be in a gene not yet described. Future studies will help investigate the roles of these genes as well as others in oxygen sensing, chromatin maintenance, and PI3K pathways.

Cardiac diseases may be overlooked during routine surveillance because chest imaging is usually performed with computed tomography, which has low sensitivity for detecting intracardiac tumors. Unrecognized delayed cardiac metastases can cause significant complications, such as syncope, heart failure, arrhythmia, hemodynamic instability, and myocardial infarction. Furthermore, late lesions may not be resectable. RCC at increased risk of cardiac metastasis can be managed with routine cardiac surveillance (echocardiogram).

## Conclusion

This single case of isolated cardiac metastasis from ccRCC that had genomic testing conducted is associated with the *VHL* V165G mutation. Further genomic characterization of cases with cardiac metastases may provide clues regarding unique mutations. Screening for the *VHL* V165G mutation is recommended in patients with delayed spread of RCC to the heart.
